# Optimization of Nonlinear Lamb Wave Detection System Parameters in CFRP Laminates

**DOI:** 10.3390/ma14123186

**Published:** 2021-06-09

**Authors:** Zhenhua Yin, Ying Tie, Yuechen Duan, Cheng Li

**Affiliations:** School of Mechanical and Power Engineering, Zhengzhou University, Science Road 100, Zhengzhou 450001, China; yinzhenhua@gs.zzu.edu.cn (Z.Y.); tieying@zzu.edu.cn (Y.T.); duanyc1984@zzu.edu.cn (Y.D.)

**Keywords:** CFRP laminates, response surface methodology, nonlinear Lamb wave, fundamental wave, second harmonic

## Abstract

Carbon fiber reinforced polymer (CFRP) laminates, as unique multifunctional materials, are widely applied in various aircraft, such as airliners, fighter planes, and space shuttles. To ensure aircraft safety during the production and application of CFRP laminates, it is necessary to improve the accuracy of nonlinear Lamb wave nondestructive testing to assess the damage in CFRP laminates caused by impact, high temperature, friction, corrosion, etc. In this study, the accuracy of nonlinear ultrasonic nondestructive testing was found to highly depend on the cycle number, output level and gain of the nonlinear ultrasonic detection system. Based on a single-factor experiment that considered the cycle number, output level, and gain of the amplifier as independent variables, a regression analysis was carried out on the fundamental wave amplitude value (A_1_) and second harmonic amplitude value (A_2_). Two response surface surrogate models were established to improve the accuracy of nonlinear Lamb wave nondestructive testing and to optimize the detection system parameters. The response surface models were verified via an analysis of variance (ANOVA), significance tests and an error statistical analysis. The results revealed the significant influence of these three factors on A_1_ and A_2_. Optimization of the response surface was achieved at eight cycles, an output level of 42 and a gain of 32 dB. Moreover, the nonlinear ultrasonic detection system achieved good operational stability, high accuracy and reliability under the above optimal parameter conditions. This approach provides scientific guidance for the accurate assessment of CFRP laminate damage.

## 1. Introduction

Carbon fiber reinforced polymer (CFRP) laminates are extensively applied in the electronics, military, and aerospace fields due to their excellent mechanical properties [1,2,3,4]. One of CFRP laminates’ critically important characteristics is an unusually high anisotropy [5,6]. Although CFRP laminates have many advantages, their production process is complex, and damage, such as fracturing, delamination and debonding, inevitably occurs during the application process [7,8,9]. Especially for aircraft composite structures during service, the environment faced is extremely complicated and quite harsh, and it is easily affected by various conditions, such as hail strikes, bird strikes, lightning strikes, and tool drops [10,11]. Damage poses a great potential danger to the safety of the material structure. Additionally, the frequency dispersion of CFRP laminates is significant, which prevents their applications in conventional nondestructive detection methods. Therefore, the development of new detection equipment and methods is necessary to identify manufacturing defects, control the quality of the final product, and ensure the safety of personnel.

In recent years, ultrasonic nondestructive detection has been widely adopted in the assessment of the safety of material structures. According to the different mechanisms of damage interaction, ultrasonic detection technology is usually classified into linear and nonlinear techniques [12,13]. Compared to linear ultrasonic detection, nonlinear Lamb waves have attracted much attention in the material characterization and detection of barely visible damage due to their higher sensitivity [14,15]. The method of detection and evaluation that involves nonlinear ultrasonic nondestruction mainly capitalizes on signal characteristics in the frequency domain to discriminate defects, which overcomes the defects of traditional ultrasonic detection techniques [16,17,18,19]. Moreover, nonlinear Lamb waves are almost nondispersive in anisotropic media so that different harmonics effectively interact in the process of wave propagation.

Nonlinear Lamb waves detect not only visible damage, such as external cracks, but also invisible damage, such as internal delamination and bonding defects, in CFRP laminates [20,21]. Therefore, nonlinear Lamb wave-based higher-harmonic detection methods have increasingly been adopted by researchers to evaluate the structural safety of composite materials [22,23]. Tie et al. [24] investigated the impact damage to CFRP laminates under different impact energies via finite element and experimental methods based on the higher harmonics of nonlinear Lamb waves. This work proposed an integrated numerical model to study the interaction relationship between nonlinear Lamb wave propagation and low-velocity impact damage. Yang et al. [25] experimentally and theoretically analyzed the second harmonic signal generated by the nonlinear interaction between nonlinear Lamb wave propagation and fatigue cracks in materials and demonstrated that the three-dimensional (3D) finite element method could properly predict the double-frequency signal due to the material contact nonlinearity that occurs at fatigue cracks. Carboni et al. [26] investigated a structural health monitoring approach based on nonlinear Lamb wave detection, which matched the out-of-phase driving of low-frequency piezoceramic transducers. Via the design of an experimental statistical method that considered the pulse echo and pitch capture configurations of piezoceramic transducer (PZT) sensors, several factors and their interactions effectively affected the detection of delamination damage. Although some of the existing studies have mainly focused on the application of nonlinear ultrasonic nondestructive testing for damage (e.g., cracks, delamination, and impact) assessment in CFRP laminates. However, there are still few studies on the optimization of nonlinear ultrasonic detection-related parameters in CFRP laminates.

To improve the precision and application of nonlinear ultrasonic detection, the extraction of a suitable fundamental wave amplitude value (A_1_) and second harmonic amplitude value (A_2_) is very important and difficult, because both A_1_ and A_2_ are affected by several factors and should be considered simultaneously, including noise interference with the instrument, filter use, amplitude of the driving voltage, and coupling with media. To accurately extract nonlinear signals to determine the state of material characterization, harmonic interference induced by the measuring instrument or random factors should be reduced to optimally simplify the frequency components of the transmitted signal for proper setting and selection. Therefore, it is necessary to study A_1_ and A_2_ under different conditions to better understand the effect of various factors (e.g., cycle number, output level, and gain) on the nonlinear ultrasonic detection system stability. The response surface method (RSM) is an effective statistical method [27] that relies on the reasonable design of experiment (DOE) method [28,29] to obtain certain data through tests, applies multiple quadratic regression equations to fit a functional relationship between the factors and response values, and determines the optimal process parameters through the analysis of regression equations to solve multivariable problems. While reducing the number of experiments, damage to the materials can be predicted, which greatly reduces costs. The RSM mainly includes the Box–Behnken design, central composite design (CCD), full factorial design, and Plackett–Burman design. Among these approaches, CCD comprises three parts, namely, a 2*^n^* (*n* is the factor) full factorial design, an axis point design, and zero level of the center point repetition test. The second-order polynomial of each influencing factor is considered to predict its effect on the evaluation index. The CCD method expands the design space and provides sample data regarding the response surface approximation model, which yields the advantages of a simple design, fewer trials, and good predictability. CCD has been widely adopted by researchers because it facilitates time, money, and manpower reduction. In addition, the RSM involving statistical optimization overcomes the shortcomings of traditional methods and examines the relationship between the independent variables via the fitting of quadratic polynomial regression equations. Therefore, this method has been widely applied in the experimental design and optimization of wastewater treatment, organic synthesis, food science, and other fields [30,31]. The detection-related parameters need to establish RSM model to be optimized and obtain their optimal detection conditions because the inaccuracy of nonlinear Lamb wave detection results for damage of CFRP laminates in aircraft structures. However, to the best of our knowledge, few studies have been performed to explore A_1_ and A_2_ by optimizing the conditions of nonlinear Lamb wave detection system parameters using the DOE method.

In this study, based on different parameter settings in the RAM-5000 SNAP nonlinear ultrasonic detection system, according to the CCD design principle of the RSM, the experimental conditions of the nonlinear ultrasonic nondestructive testing amplitudes A_1_ and A_2_ were optimized. Mathematical regression models of A_1_ and A_2_ were established by the RSM with the cycle number, output level and gain as the independent variables, and the average values of A_1_ and A_2_ retrieved from three parallel tests were adopted as the response value. The optimal experimental conditions were explored by RSM, and a theoretical basis for further studies on nonlinear ultrasonic detection was provided. This simple and reliable evaluation method of material damage may greatly improve the accuracy of online, dynamic, and real-time aircraft structural health monitoring (SHM) systems of aircraft composites during operation, and achieve a qualitative leap in safety monitoring and the performance of engineering structures. The rest of this paper is arranged as follows. Section 2 introduces the experimental study, including the RAM-5000 SNAP nonlinear ultrasonic detection system setup, RSM model experimental design, calculation of the ultrasonic nonlinear parameter and relative error. Section 3 describes the nonlinear ultrasonic detection method, the effect of single-factor experiments on A_1_ and A_2_, and the response surface model analysis. Section 4 presents the results and discussion and proposes the optimal experimental design parameters. Finally, this paper ends with a conclusion.

## 2. Experimental Study

### 2.1. Experimental Setup

**Figure 1:**
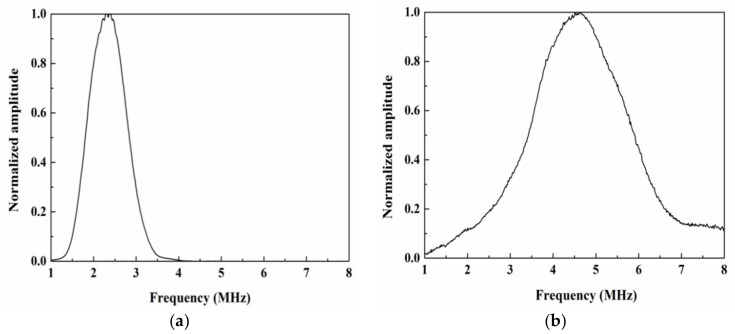
Frequency response range of the (**a**) excitation and (**b**) receiving transducers.

A frequency-sweep analysis of the transducer is conducted to obtain its frequency response and select the appropriate excitation frequency. Frequency-sweep tests are carried out with superheterodyne technology and the phase-sensitive detector of a RAM-5000 SNAP system (RITEC Inc., Warwick, RI, USA), with the nonlinear Lamb wave signal chosen as the integral window. In this experiment, two ultrasonic transducers, one with a central frequency of 2.25 MHz (Olympus NDT Panametrics, A542S, Olympus, WA, USA) and the other with a central frequency of 5 MHz (Olympus NDT Panametrics, V543, Olympus, WA, USA), are applied as excitation and receiving transducers, respectively. Figure 1 shows the frequency response range of these two transducers. The excitation transducer is a narrowband longitudinal ultrasonic transducer, and the receiver is a broadband longitudinal ultrasonic transducer.

The damaged material tested in the experiment was a T9001 CFRP laminate (Weihai Guangwei Composites Co., Ltd., Weihai, China) cut with a high-pressure water jet cutter (Anhui Aoyu CNC Technology Co., Ltd., Chuzhou, China). The CFRP laminate consists of 24 layers with a total thickness of 3.6 mm and linear dimensions of 360 × 250 mm^2^. The layer stacking sequence is [45/0/−45/90]_3s_, and the single-layer thickness is 0.15 mm.

**Figure 2:**
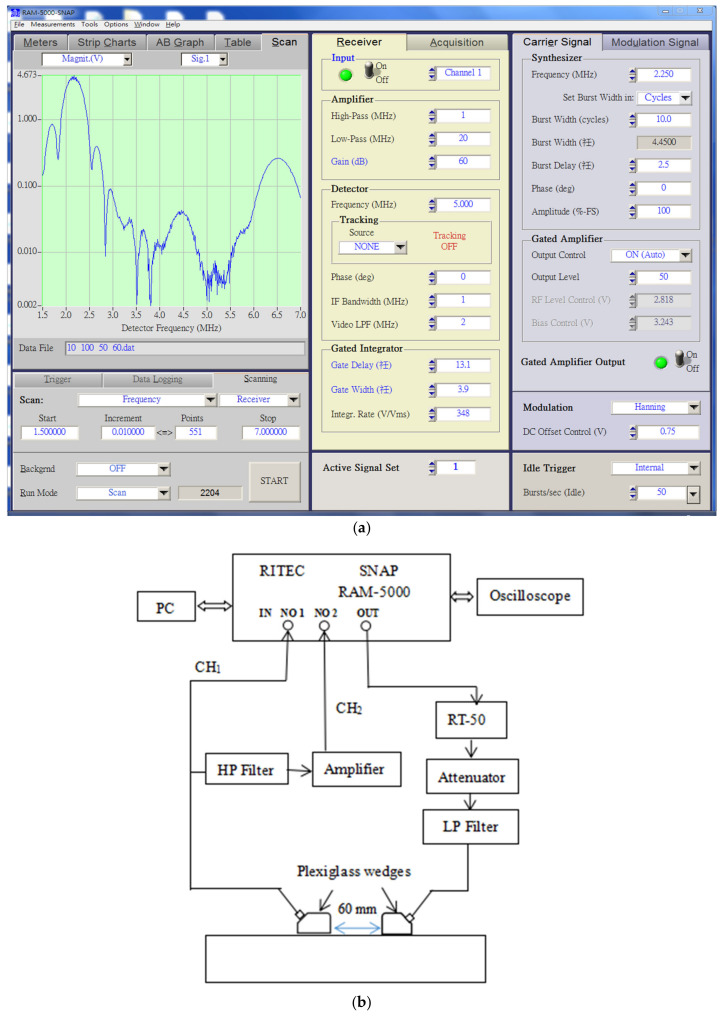

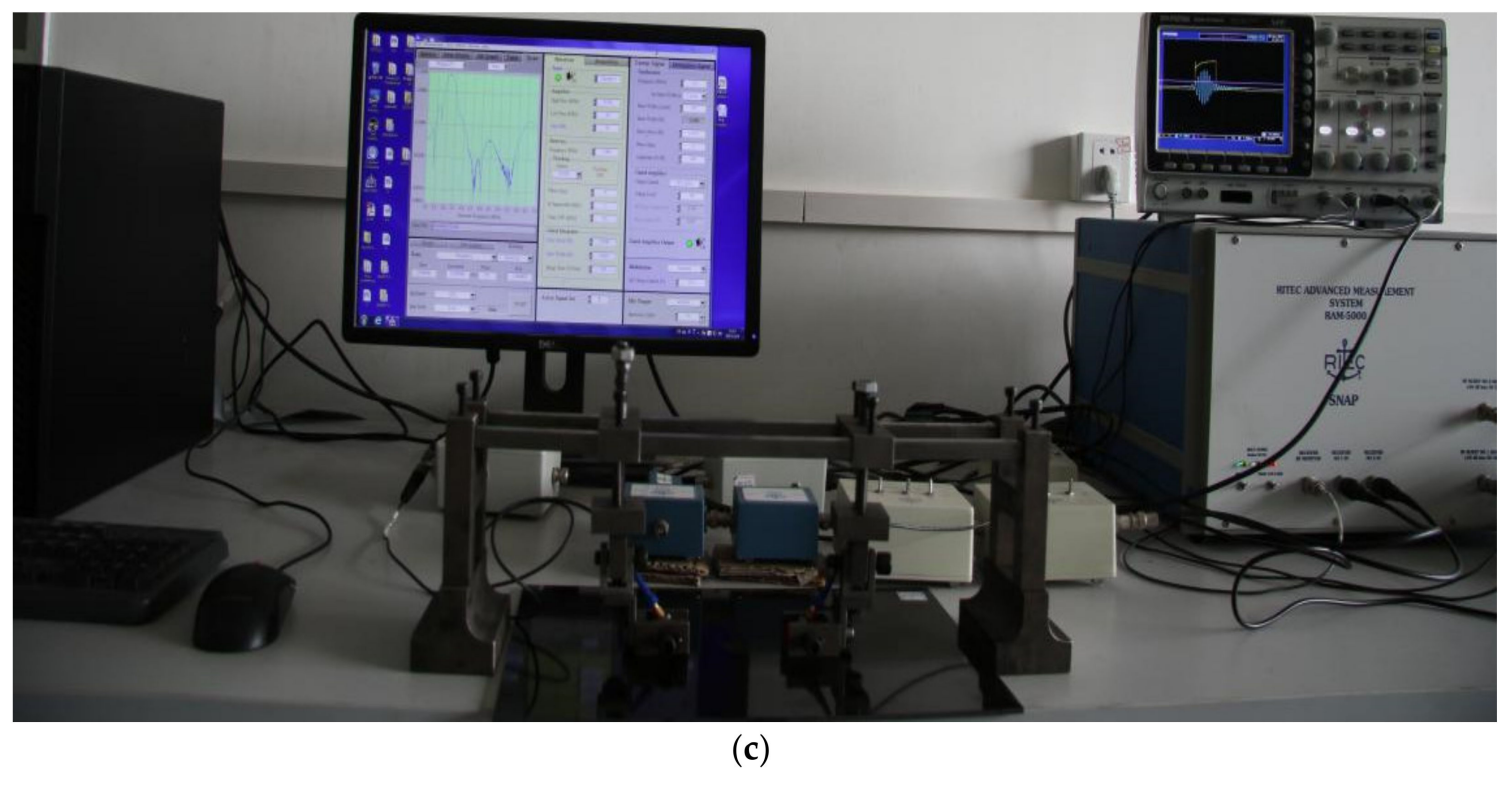
Nonlinear ultrasonic detection system: (**a**) Computer-controlled RAM-5000 SNAP ultrasonic detection system parameter settings, (**b**) block diagram of the experimental system setup, and (**c**) photograph of the experimental system.

A RAM-5000 SNAP nonlinear ultrasonic detection system is employed to generate high-power tone burst signals [32], and the frequency, cycle number, output level and gain of the signals can be conveniently and flexibly set through a computer-controlled panel, as shown in Figure 2a. A block diagram of the setup is shown in Figure 2b. Its key component, the RAM-5000 SNAP detection system, emits sequences of tone burst cycles attenuated by 4 dB. After the 2.25 MHz low-frequency filter group removes any high-frequency interference generated by the device, the transducer with a center frequency of 2.25 MHz generates ultrasonic Lamb waves that enter the samples. The broadband transducer (5 MHz) is employed to receive the propagated signal via the fundamental wave channel (CH_1_) and second harmonic channel (CH_2_). After 4.5 MHz high-pass filtering and 20 dB preamplification, the signal is received at CH_2_, after which it enters the RAM-5000 SNAP system for signal extraction and processing. A photograph of the experimental test system is shown in Figure 2c.

Nonlinear Lamb waves are excited and received in the CFRP laminates through two longitudinal ultrasonic transducers mounted on Plexiglas wedges at an angle of 45°. The optimal distance between the Plexiglas wedges is 60 mm. Mount 7501 high-vacuum silicone grease is applied as the coupling agent, and the excitation and receiving transducers are positioned along the same straight line. A special fixture is employed to ensure full coupling between the sensors and samples during detection under constant pressure.

In this study, three main parameter settings that cycle numbers ranging from 5 to 14, the output level ranging from 30 to 55 and the gain ranging from 28 to 50 are considered. The nonlinear Lamb wave detection system is used to measure the sample response under the same conditions three times; then, the average value is taken as A_1_ and A_2_. To overcome the effect of random noise generation, the relative error of the repeated measurement is set below 1.5%.

### 2.2. Response Surface Model Experimental Design

The RSM model relies on CCD to optimize the most effective variables under the parameter setting conditions of the RAM-5000 SNAP nonlinear ultrasonic detection system and to study their mutual relationships. Based on the one-factor experimental results, the number of cycles (*x*_1_), output level (*x*_2_), and gain (*x*_3_) are chosen as the three independent variables. A_1_ and A_2_ averaged over three parallel tests are selected as the response values for the regression analysis with the RSM model considering 3 factors and 5 levels. Each parameter of this model was set according to various five-level codes (−2, −1, 0, 1, and 2), and the detailed range and level results of the experimental design factors are listed in Table 1.

In the regression analysis equation, the actual values are converted into coded values as follows:

(1) $$X_{i}=(x_{i}-x_{0})/\Delta x$$

where *X_i_* denotes the coded value of the independent variable, *x_i_* represents the actual value of the independent variable at the *i*th level, *x*_0_ indicates the actual value of the independent variable at the center point, and Δ*x* is the step change value.

The experimental data of CCD are fitted to a second-order polynomial regression Equation (2) via the RSM.

(2) $$\begin{array}{l} y=a+a_{1}x_{1}+a_{2}x_{2}+a_{3}x_{3}+a_{12}x_{1}x_{2}+a_{13}x_{1}x_{3}+a_{23}x_{2}x_{3}+a_{11}x_{1}^{2}+a_{22}x_{2}^{2}+a_{33}x_{3}^{2}\\ \;=\left[\begin{array}{cccc} 1 & x_{1} & x_{2} & x_{3} \end{array}\right]\;\left[\begin{array}{cccc} a & \frac{a_{1}}{2} & \frac{a_{2}}{2} & \frac{a_{3}}{2}\\ \frac{a_{1}}{2} & a_{11} & \frac{a_{1}{}_{2}}{2} & \frac{a_{1}{}_{3}}{2}\\ \frac{a_{2}}{2} & \frac{a_{1}{}_{2}}{2} & a_{22} & \frac{a_{2}{}_{3}}{2}\\ \frac{a_{3}}{2} & \frac{a_{1}{}_{3}}{2} & \frac{a_{2}{}_{3}}{2} & a_{33} \end{array}\right]\;\left[\begin{array}{c} 1\\ x_{1}\\ x_{2}\\ x_{3} \end{array}\right] \end{array}$$

where y is the predicted response value; $x_{1}$, $x_{2}$, and $x_{3}$ are the actual values of the independent variables; a is a constant coefficient; $a_{1}$, $a_{2}$, and $a_{3}$ are the linear-effect coefficients; $a_{11}$, $a_{22}$, and $a_{33}$ are the squared effect coefficients; $a_{12}$, $a_{13}$, and $a_{23}$ are the interaction effect coefficients. The results are analyzed graphically and statistically with Design-Expert software to determine the interaction relationship among the above three factors to finally obtain the RSM model.

### 2.3. Calculation of the Ultrasonic Nonlinear Parameter and Relative Error

Based on the isotropic assumption of nonlinear acoustic theory, in the early stage of material damage, the distribution in different directions has less effect on the mechanical properties of the material. Therefore, it is assumed that material damage does not lead to anisotropy of the material. For this reason, the anisotropy analysis of material is summarized in isotropic to study.

In the case of small strain, if attenuation during propagation is neglected. Consider the one-dimensional plane wave in the *xy* plane. Assuming that the wave propagates only along the *x*-axis, the particle displacement u is related only to the position x and time t. The wave equation is obtained, as follows:

(3) $$\rho \frac{\partial^{2}u}{\partial t^{2}}=\frac{\partial \sigma }{\partial x}$$

where ρ is the density and σ is the stress.

Ultrasonic Lamb waves are distorted due to their nonlinear interaction with the composite material during propagation, which may generate high-order harmonic components. Based on the physical mechanism, the wave beam response phenomenon is caused by the nonlinearity of the material elastic behavior. According to the nonlinear Hooke law [33], the interaction between σ and strain ε is as follows:

(4) σ=Εε(1+βε+⋯)

where Ε is Young’s modulus of the material; and β is the absolute nonlinear elastic constant of the second-order [34], which is due to the wave interaction with the imperfect interfaces causing a local change in the stiffness at that region.

According to perturbation approximation theory [35], the approximate solution of the one-dimensional nonlinear wave Equation (3) in an isotropic solid is expressed in Equation (5).

(5) $$\beta =\frac{8}{k^{2}x}\frac{A_{2}}{A_{1}^{2}}$$

where A_1_ is the amplitude at ω, A_2_ is the amplitude at 2ω, *k* = ω/*v* (*k* is the wavenumber, which is proportional to the square root of Young’s modulus, *v* is the ultrasonic phase velocity), and *x* is the Lamb wave propagation distance.

When the detection conditions remain the same, *k* and *x* should be kept constant. Therefore, Equation (5) can be simplified, then the relative acoustic nonlinear parameter $\beta^{\prime }$ is introduced [36,37] and represents a generalized parameter associated with the nonlinear properties of Lamb waves.

(6) $$\beta^{\prime }\propto \frac{A_{2}}{A_{1}^{2}}$$

$\beta^{\prime }$ is a quantitative indicator that can be used to characterize the microdamage degree in CFRP laminates in the following discussion. When $\beta^{\prime }$ is larger, the internal damage in the CFRP laminate is more serious, and vice versa.

The absolute error is considered to estimate the error range between the actual and predicted response values of A_1_ and A_2_, and the relative error is considered to determine the reliability of the response surface model according to Equations (7) and (8), respectively.

(7) $$\Delta =L-{L^{\prime }}_{0}$$

(8) $$\delta =\frac{\Delta }{{L^{\prime }}_{0}}\times 100\%$$

where *L* denotes the actual value of A_1_ and A_2_, ${L^{\prime }}_{0}$ represents the predicted values of A_1_ and A_2_, Δ is the absolute error, and *δ* is the relative error.

## 3. Experimental Work

### 3.1. Nonlinear Lamb Wave Detection

According to the setup of the nonlinear ultrasonic detection system, as shown in Figure 2b, the experiment adopts the sensor arrangement method [38], where one transducer excites and the other transducer receives components to measure A_1_ and A_2_, respectively. To minimize the generation of harmonics, the transmitted signal should not overlap the received signal during propagation in the sample. Therefore, we apply sine pulse excitation in the experiment and record the received signal with an oscilloscope, conduct additional Hanning window debugging, and observe the oscilloscope waveform to ensure that the measured samples generate a single-mode Lamb wave. The fast Fourier transform (FFT) is utilized to convert the signals from a time-domain representation to the frequency domain to be stored on the computer.

**Figure 3:**
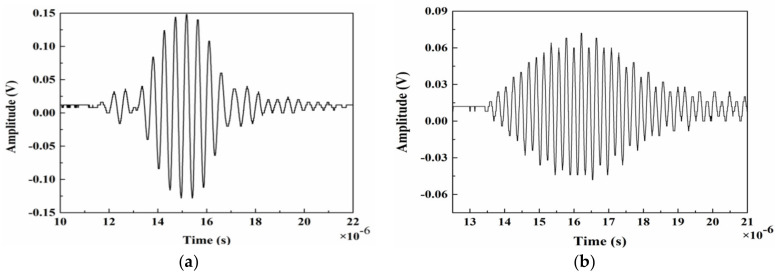
Lamb wave time-domain signal recorded by the oscilloscope: (**a**) Fundamental wave and (**b**) second harmonic.

**Figure 4:**
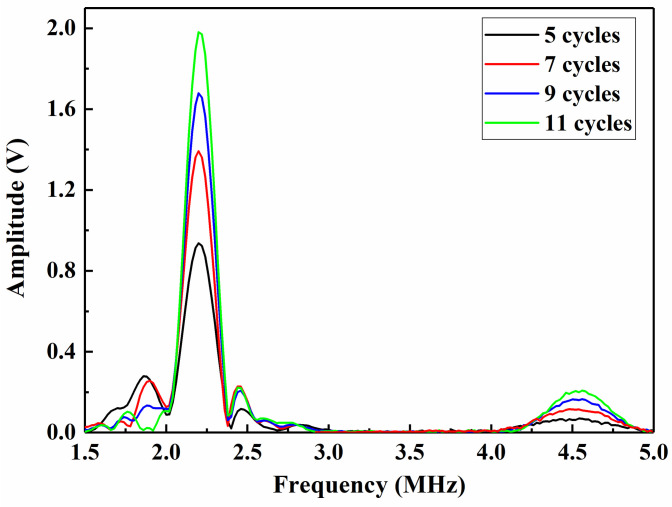
Frequency-domain signal during various cycles.

At an excitation frequency of 2.25 MHz and a cycle number of 9, the time-domain signals of Lamb waves are recorded with the oscilloscope, as shown in Figure 3. The frequency-domain signal during the various cycles is shown in Figure 4. Then, A_1_ and A_2_ are extracted, and with an increasing number of cycles, the amplitude continues to increase, and the signal is significantly enhanced.

### 3.2. Single-Factor Experiment

**Figure 5:**
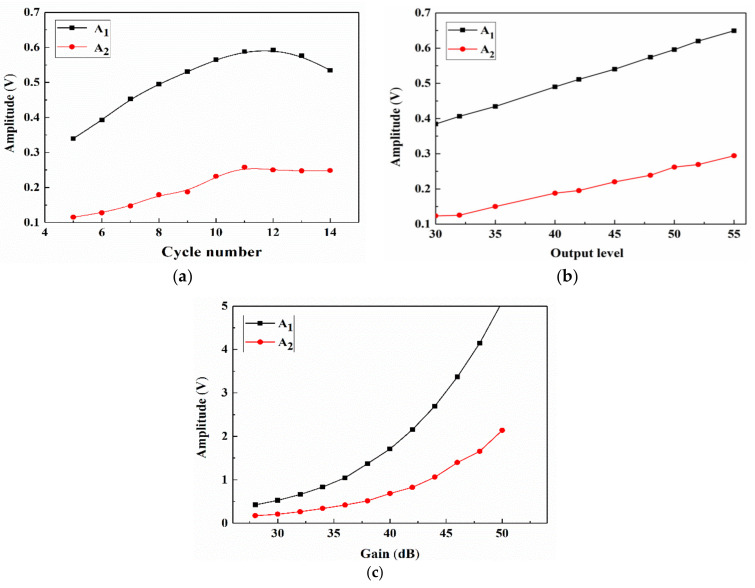
Single-factor experimental effect of the (**a**) cycle number, (**b**) output level, and (**c**) gain on A_1_ and A_2_.

Before the RSM analysis, the factors and levels of the experiment should be selected according to the range of the individual factors. Under the same conditions, only one independent variable is adjusted, and its effect on the nonlinear amplitude is determined, as shown in Figure 5. If the number of cycles is small, then the launch energy is low, which makes it difficult to obtain harmonics at the receiving end. If the number of cycles is large, the wave of the first cycle propagates through the sample and is reflected, thereby interfering with the newly excited wave. Therefore, the measurements could be inaccurate. The effect of the cycle number on the amplitude is shown in Figure 5a. With an increasing number of cycles, the values of A_1_ and A_2_ also exhibit a rapid increase to reach maximum values at 11 cycles followed by a decrease. A_1_ and A_2_ mainly increase because of the increase in number of cycles and launch energy. However, since the test sample is thinner, nonlinearity of the pulse strings occurs due to overlap, and the trailing effect of the received signal should be minimized. The data fluctuate within the range from 7 to 11 pulse strings, which corresponds to a relatively small cycle number and a relatively stable measurement environment. Figure 5b,c shows that both the output level and gain indicate amplification of the received signal. With an increasing signal, the amplitude value continuously increases. However, an excessive signal leads to an excessive voltage, which could damage the device, and the signal could become too large to receive the excessive amplitude value, which easily leads to distortion. Thus, to guarantee the signal stability, the step size of the output level and gain should be gradually increased.

### 3.3. Response Surface Model Analysis

**Figure 6:**
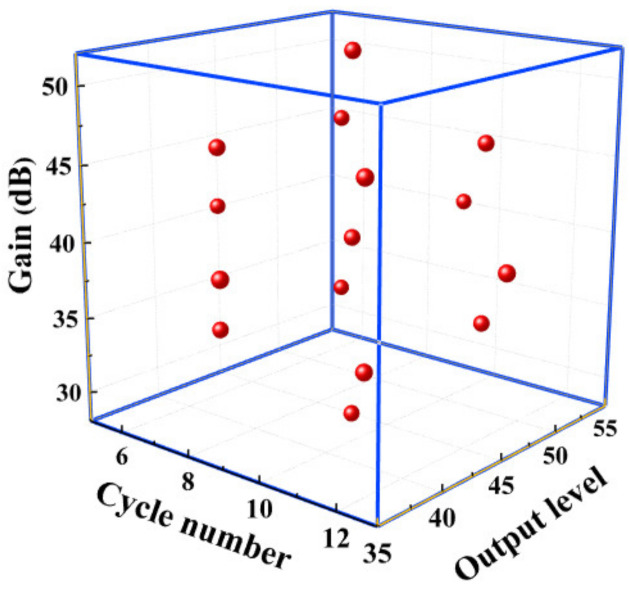
DOE space of the three design parameters and the selected training points.

The number of sample points randomly selected via CCD in the experimental design space is given by Equation (9) [39]:

(9) $$N=2^{n}+2n+\lambda_{0}$$

where *N* is the total number of experiments, *n* is the number of independent variables, and $\lambda_{0}$ is the number of repeated experiments at the center point. Regarding this design, the factor number is 3 and $\lambda_{0}$ is 6. Therefore, a total of 20 groups of training points are selected, and the DOE space of the three design parameters is constructed, as shown in Figure 6. The experimental results of CCD are listed in Table 2.

An analysis of variance (ANOVA) [40] was employed to assess the significance and accuracy of the response surface model, and the results of the fitted quadratic polynomial regression model to response surface values A_1_ and A_2_ are listed in Table 3. ANOVA is also referred to as an F-test, and the F-value at the corresponding significance level was considered to assess the difference between sample points. When the F-value is higher, the fitted equation is more significant, and the fit is better. The *p*-value is the probability value under the corresponding F value, which reflects the conditional probability of the occurrence of extreme result D under the original hypothesis H (i.e., prob (D/H)). High F-values and low *p*-values ensure the significance of the RSM model [41]. *p*-values of less than 0.05 demonstrate that the RSM model is significant, and a good fitting accuracy ensures the subsequent response surface design of the approximation model. Consequently, the model *p*-values for A_1_ and A_2_ are below 0.0001, which suggests that both RSM models are highly significant. Regarding the two models of A_1_ and A_2_, the F-values are 5498.52 and 353.71, respectively, which suggest that they are significant. The lack-of-fit F-values of 2.01 and 2.44, and the lack-of-fit *p*-values of 0.2314 and 0.1752 for A_1_ and A_2_, respectively, suggest that the two models are not significant (*p* > 0.05) relative to the pure error. A nonsignificant lack of fit is good, which indicates that the fitting degree of the two models is suitable, and the test error is small. In this case, *x*_1_, *x*_2_, *x*_3_, *x*_1_^2^, *x*_3_^2^, *x*_1×3_, and *x*_2×3_ of response A_1_ and *x*_1_, *x*_2_, *x*_3_, *x*_1×3_, *x*_2×3_, and *x*_3_^2^ of response A_2_ are highly significant (*p* < 0.01), and these values indicate a great influence on responses A_1_ and A_2_, rather than simple linear relationships.

The error statistical analysis results of the regression equations are provided in Table 4. The coefficient of variation (C.V.) values are 1.39% and 5.11% for A_1_ and A_2_, respectively, which are less than 10%, which indicates a high accuracy and reliability of the two models. Adeq. Precision reflects the signal-to-noise ratio, and this value should be higher than 4. Regarding the two models, the ratios of 286.028 and 73.321 are obtained, which suggests that the signal level is adequate. The two response surface models can be employed to navigate the DOE space. Furthermore, the R-squared values are 0.9998 and 0.9969 for A_1_ and A_2_, respectively. When the multiple correlation coefficient R^2^ is higher, the correlation is better. Based on Table 4, regarding the two models, the obtained R^2^ values are greater than 0.99, which indicates that 99% of the total variation in A_1_ and A_2_ can be attributed to the experimental variables, and the experimental values are highly similar to the predicted values. Additionally, the R_pred_^2^ values are consistent with the R_adj_^2^ values. This suggests that all empirical models contain significant terms. According to the statistical analysis of the above regression equations, the regression equation model is the most suitable for this study.

The final second-order polynomial equations in terms of the coded factors are

(10) $$A_{1}=\left[\begin{array}{cccc} 1 & X_{1} & X_{2} & X_{3} \end{array}\right]\left[\begin{array}{cccc} 0.39 & 0.03 & 0.0215 & 0.16\\ 0.03 & -0.012 & 2.1935\times 10^{-3} & 0.0245\\ 0.0215 & 2.1935\times 10^{-3} & 3.182\times 10^{-5} & 0.015\\ 0.16 & 0.0245 & 0.015 & 0.11 \end{array}\right]\left[\begin{array}{c} 1\\ X_{1}\\ X_{2}\\ X_{3} \end{array}\right]$$

(11) $$A_{2}=\left[\begin{array}{cccc} 1 & X_{1} & X_{2} & X_{3} \end{array}\right]\left[\begin{array}{cccc} 0.25 & 0.0155 & 0.0145 & 0.095\\ 0.0155 & -6.927\times 10^{-3} & 0.008 & 0.0085\\ 0.0145 & 0.008 & -1.740\times 10^{-3} & 0.0095\\ 0.095 & 0.0085 & 0.0095 & 0.06 \end{array}\right]\left[\begin{array}{c} 1\\ X_{1}\\ X_{2}\\ X_{3} \end{array}\right]$$

The final second-order polynomial equations in terms of the actual factors are

(12) $$A_{1}=\left[\begin{array}{cccc} 1 & x_{1} & x_{2} & x_{3} \end{array}\right]\left[\begin{array}{cccc} 5.5355 & -0.0506 & -0.0178 & -0.1320\\ -0.0506 & -2.9233\times 10^{-5} & 2.1937\times 10^{-4} & 2.0536\times 10^{-3}\\ -0.0178 & 2.1937\times 10^{-4} & 1.2727\times 10^{-5} & 0.5031\times 10^{-3}\\ -0.1320 & 2.0536\times 10^{-3} & 0.5031\times 10^{-3} & 2.9346\times 10^{-3} \end{array}\right]\left[\begin{array}{c} 1\\ x_{1}\\ x_{2}\\ x_{3} \end{array}\right]$$

(13) $$A_{2}=\left[\begin{array}{cccc} 1 & x_{1} & x_{2} & x_{3} \end{array}\right]\left[\begin{array}{cccc} 3.2970 & -0.04171 & -0.01379 & -0.07179\\ -0.04171 & -1.7318\times 10^{-3} & 0.7956\times 10^{-3} & 0.7286\times 10^{-3}\\ -0.01379 & 0.7956\times 10^{-3} & -6.9591\times 10^{-5} & 3.1729\times 10^{-4}\\ -0.07179 & 0.7286\times 10^{-3} & 3.1729\times 10^{-4} & 1.6597\times 10^{-3} \end{array}\right]\left[\begin{array}{c} 1\\ x_{1}\\ x_{2}\\ x_{3} \end{array}\right]$$

**Figure 7:**
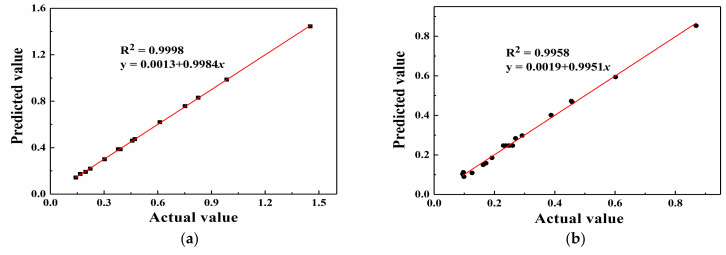
Comparison of the predicted and actual values: (**a**) A_1_ and (**b**) A_2_.

**Figure 8:**
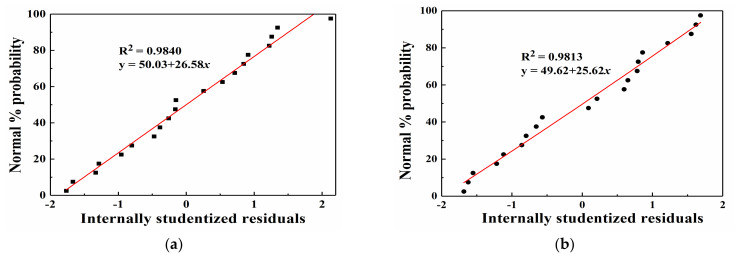
Normal probability distribution of the residuals: (**a**) A_1_ and (**b**) A_2_.

**Figure 9:**
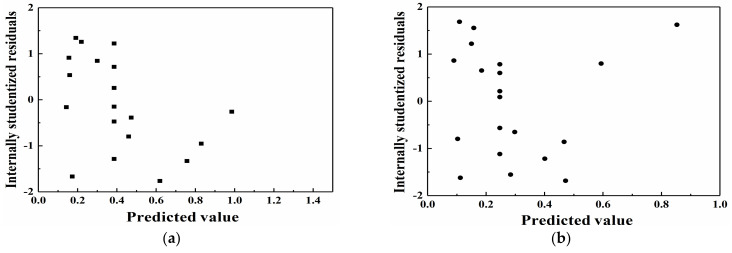
Distribution of the predicted values and internally studentized residuals: (**a**) A_1_ and (**b**) A_2_.

The internally studentized residuals are the values obtained by dividing the residuals by their standard deviations, which are considered to intuitively assess whether the assumption that the error terms obey a normal distribution is true. If the assumption holds true, then the distribution of the internally studentized residuals should also obey a normal distribution. The internally studentized residuals are the statistics of the abnormal point test. Figure 7, Figure 8 and Figure 9 show a comparison of the predicted and actual values, the residual normal probability distribution, and the internal residual and predicted value distributions. The comparison of the experimental and predicted values reveals that a high degree of similarity exists between the actual and predicted values. Furthermore, the normal probability distribution of the residuals exhibits near-linearity, and irregular distributions of the internally studentized residuals and predicted values are obtained. Based on Figure 7, Figure 8 and Figure 9, the response surface models of A_1_ and A_2_ attain good adaptability in nonlinear ultrasonic nondestructive detection.

## 4. Results and Discussion

**Figure 10:**
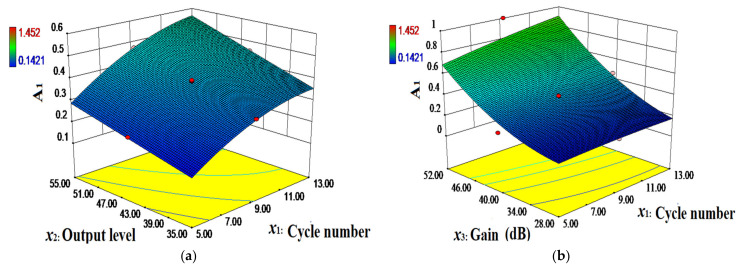

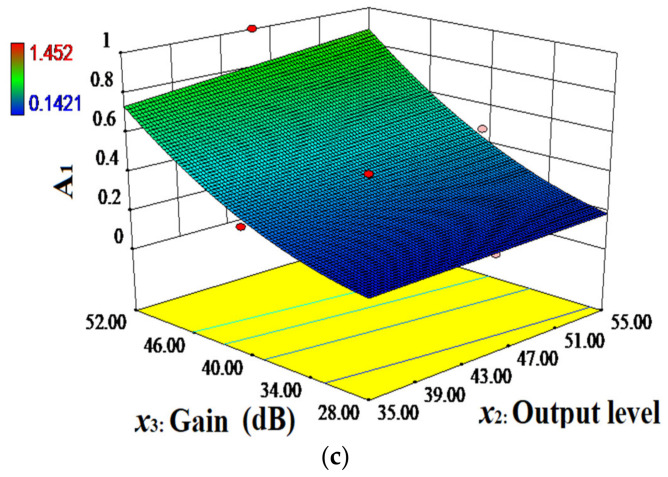
Three-dimensional (3D) response plots: (**a**) Cycle number (*x*_1_) and output level (*x*_2_), (**b**) cycle number (*x*_1_) and gain (*x*_3_), (**c**) output level (*x*_2_) and gain (*x*_3_), and their mutual interactions with A_1_.

**Figure 11:**
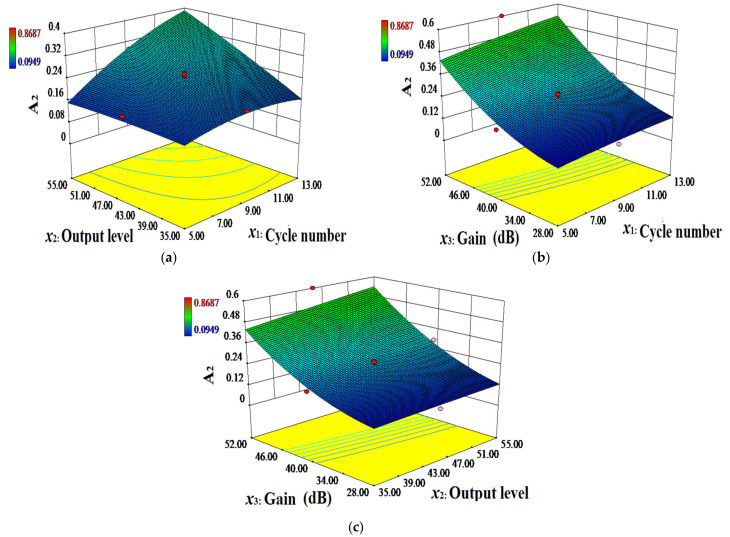
Three-dimensional (3D) response plots: (**a**) Cycle number (*x*_1_) and output level (*x*_2_), (**b**) cycle number (*x*_1_) and gain (*x*_3_), (**c**) output level (*x*_2_) and gain (*x*_3_), and their mutual interactions with A_2_.

To more intuitively analyze the effect of each factor, determine their mutual interactions with A_1_ and A_2_, and characterize the shape of the response surface function, 3D response plots are generated according to the quadratic polynomial regression equations, as shown in Figure 10 and Figure 11. These figures demonstrate the interaction effect of the independent variables with A_1_ and A_2_ when a certain factor remains fixed at the same central value.

Figure 10 and Figure 11 show that when the slope of the curve is steeper, the effect of the independent variables on the amplitude is more significant. Moreover, when the slope is smoother, the effect is less significant. The effects of the cycle number, output level, and gain on amplitudes A_1_ and A_2_ of the nonlinear ultrasonic waves are significant with steep curves. The response surface plots of the cycle number and output level on A_1_ and A_2_ exhibit open downward convex surfaces. This indicates that the response values are extremely high within the range of the experimental factors, and the optimal values of the cycle number and output level are 8~10 and 40~45, respectively. The interactions between the number of cycles and gain, and between the output level and gain impose a strong effect on the nonlinear ultrasonic wave amplitudes A_1_ and A_2_, which results in high-density contour lines. The interaction between the number of cycles and output level imposes no significant effect on nonlinear ultrasonic wave amplitude A_1_, and the contour lines are not dense. The interaction between the number of cycles and output level exerts a secondary significant effect on nonlinear ultrasound amplitude A_2_, and the contour lines are not dense, which is consistent with the variance analysis results.

Furthermore, the second-order polynomial response surface model is a conic function. Therefore, a maximum, minimum, or saddle value exists. Thus, to further determine the optimal point, we obtain the derivative with respect to the actual value of each independent variable (*x_i_*) by setting the quadratic nonlinear regression model (i.e., Equations (12) and (13)) to zero and then applying the MATLAB software to solve these equations.

The obtained equations are

(14) $$\left[\begin{array}{ccc} -5.8466\times 10^{-3} & 4.3875\times 10^{-4} & 4.1073\times 10^{-3}\\ 4.3875\times 10^{-4} & 2.5455\times 10^{-6} & 1.0063\times 10^{-3}\\ 4.1073\times 10^{-3} & 1.0063\times 10^{-3} & 5.8691\times 10^{-3} \end{array}\right]\left[\begin{array}{c} x_{1}\\ x_{2}\\ x_{3} \end{array}\right]=\left[\begin{array}{c} 0.1012\\ 0.0357\\ 0.2641 \end{array}\right]$$

The solutions of the model relative to the actual values of A_1_ are *x*_1_ = 8.2531, *x*_2_ = 43.6944, and *x*_3_ = 31.7245.

(15) $$\left[\begin{array}{ccc} -3.4636\times 10^{-3} & 1.5913\times 10^{-3} & 1.4573\times 10^{-3}\\ 1.5913\times 10^{-3} & -1.3918\times 10^{-4} & 6.3458\times 10^{-4}\\ 1.4573\times 10^{-3} & 6.3458\times 10^{-4} & 3.3193\times 10^{-3} \end{array}\right]\left[\begin{array}{c} x_{1}\\ x_{2}\\ x_{3} \end{array}\right]=\left[\begin{array}{c} 0.08343\\ 0.02759\\ 0.1436 \end{array}\right]$$

The solutions of the model relative to the actual values of A_2_ are *x*_1_ = 8.3877, *x*_2_ = 41.7520, and *x*_3_ = 31.5943.

Considering operational convenience, the simultaneous detection of A_1_ and A_2_ under the same conditions to ensure the reliability of the response surface results given the above optimized conditions is preferable. The optimal experimental parameters regarding the response surface include approximately 8 cycles, an output level of 42, and a gain of 32 dB. Under these conditions, the averaged A_1_ and A_2_ values based on the three parallel tests are 0.1502 and 0.0976, with mean errors of 0.31% and 4.85%, respectively. This work indicates that the RSM models are feasible and significant for predicting A_1_ and A_2_ under different cycle numbers, output level and gain in the nonlinear ultrasonic detection system.

To further verify that the RSM model satisfies the requirements of the amplitude, we randomly selected ten training points outside the response surface level and substituted them into Equations (12) and (13) to compare the accuracy of the predicted values and experimental data. The calculation results for the corresponding nonlinear coefficient *β*′ are listed in Table 5, and the relative errors in the response values A_1_, A_2_, and *β*′ are below 10%. The experimentally measured values match the predicted values from the regression model, which indicates that the response surface model fits the actual situation accurately and that the RSM application to the experimental parameters of the nonlinear ultrasonic amplitude is optimized. The established quadratic nonlinear regression model is accurate and effective, and the experimental fitting effect is suitable, which yields a certain practical value.

**Figure 12:**
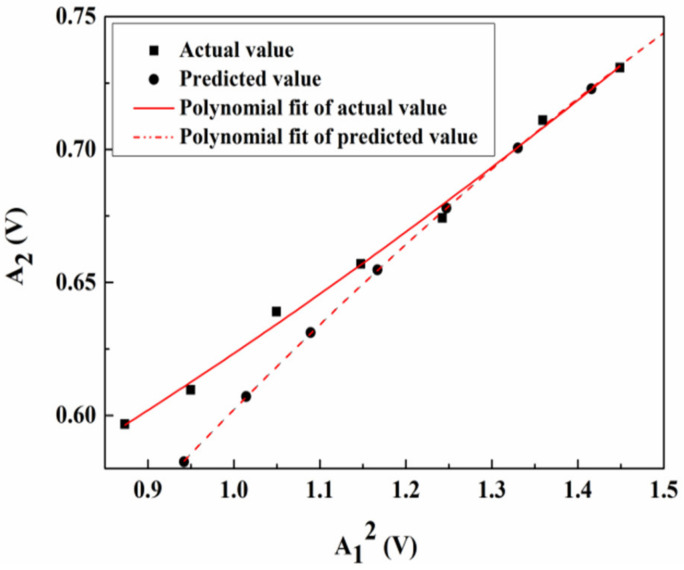
Ratio of A_2_ to A_1_^2^ with increasing excitation voltage.

Under the same conditions and within a certain range of the input voltage, the output level of the gated amplifier ranges from 36 to 48. By inputting different excitation voltages, a series of A_1_^2^ and A_2_ is generated, as shown in Figure 12. The relationship curve of A_1_^2^ and A_2_ can be obtained based on the measured data via polynomial fitting (i.e., *y*_1_ = 0.45305 + 0.12188 *x*_1_ + 0.12188 *x*_1_^2^) and the correlation coefficient is 0.9935. Compared to the predicted values, the data fitting relationship curve of the response surface equation is *y*_2_ = 0.18316 + 0.50948 *x*_2_ − 0.09045 *x*_2_^2^, and the correlation coefficient reaches 0.9997, while the slope of the linear fitting is *β*′. When the material properties and Lamb wave propagation distance remain constant, *β*′ is a constant value, which indicates that the nonlinear response of the ultrasonic detection system in the experiment is stable and does not change with the excitation signal voltage. This further confirms that the nonlinear response is not caused by the external environment but by the interaction between the ultrasonic wave and nonlinear source in the specimen.

## 5. Conclusions

Regression models that describe nonlinear ultrasonic amplitudes A_1_ and A_2_ and the relationship among the cycle number, output level, and gain were obtained via CCD. The reliability of these response surface models was verified through ANOVA, error statistical analysis and normal probability distribution determination of the residuals. The results revealed that the models were reliable and precisely predicted amplitudes A_1_ and A_2_. The influence of these three factors on A_1_ and A_2_ and the interaction between the cycle number and both the output level and gain were significant. Regarding the response surface optimization, the values of the cycle number, output level, and gain were 8, 42 and 32 dB, respectively. Under these conditions, the average A_1_ and A_2_ values based on three parallel tests were 0.1502 and 0.0976, with mean errors of 0.31% and 4.85%, respectively. To further verify that the RSM model could reflect the requirements of the amplitude, we randomly selected ten points outside the response surface level. The relative errors in A_1_, A_2_, and *β*′ were below 10%, which indicates that the application of the response surface analysis method in nonlinear amplitude ultrasonic nondestructive testing could help determine the optimal process parameters and effectively reduce the challenge of empirically setting the experimental conditions, which could represent a starting point for further studies. In addition, the RSM model may be a promising way to predict A_1_ and A_2_ among nonlinear Lamb wave detection system parameters under different conditions and to study the interaction among all factors, which suggests a method for the optimization and quantitative assessment of CFRP laminate damage. The optimized conditions determined with the RAM-5000 SNAP detection system could also be employed to detect other materials, such as metals and woven composites, and have good applicability for other detection distances. In addition, the optimized conditions could be directly adopted in the mixed-frequency detection of material damage. Furthermore, this method provides the possibility to accurately monitor the structural health of composite materials.

## Figures and Tables

**Table 1:** Experimental design factors range and levels.

Factors	Variable	Range and Coded Levels				
		−2	−1	0	1	2
Cycle number	*x*_1_	5	7	9	11	13
Output level	*x*_2_	35	40	45	50	55
Gain (dB)	*x*_3_	28	34	40	46	52

**Table 2:** Experimental results of CCD.

Run	Coded			Actual			Actual Values (V)		Predicted Values (V)		Relative Errors (%)	
	*X*_1_	*X*_2_	*X*_3_	*x*_1_	*x*_2_	*x*_3_	A_1_	A_2_	A_1_′	A_2_′	_*δ*A1_	_*δ*A2_
1	−1	1	−1	7	50	34	0.1623	0.0987	0.1602	0.0939	1.31	5.07
2	1	−1	1	11	40	46	0.8260	0.4583	0.8298	0.4668	−0.46	−1.82
3	1	−1	−1	11	40	34	0.1598	0.1086	0.1562	0.0997	2.30	8.97
4	1	1	−1	11	50	34	0.1965	0.1627	0.1912	0.1520	2.77	7.06
5	2	0	0	13	45	40	0.4574	0.2695	0.4603	0.2802	−0.63	−3.83
6	−1	1	1	7	50	46	0.7521	0.4546	0.7574	0.4673	−0.70	−2.72
7	0	0	0	9	45	40	0.3905	0.2507	0.3863	0.2468	1.09	1.60
8	1	1	1	11	50	46	0.9845	0.6019	0.9855	0.5953	−0.10	1.11
9	0	0	0	9	45	40	0.3787	0.2386	0.3863	0.2468	−1.97	−3.31
10	0	0	0	9	45	40	0.3878	0.2488	0.3863	0.2468	0.39	0.83
11	0	2	0	9	55	40	0.4716	0.2918	0.4730	0.2984	−0.30	−2.20
12	−1	−1	1	7	40	46	0.6122	0.3880	0.6192	0.4025	−1.13	−3.60
13	0	0	0	9	45	40	0.3854	0.2596	0.3863	0.2468	−0.23	5.20
14	0	0	−2	9	45	28	0.1674	0.1066	0.1734	0.1155	−3.46	−7.70
15	−1	−1	−1	7	40	34	0.1421	0.0949	0.1427	0.1053	−0.42	−9.85
16	0	0	0	9	45	40	0.3835	0.2300	0.3863	0.2468	−0.72	−6.79
17	0	0	0	9	45	40	0.3935	0.2567	0.3863	0.2468	1.86	4.03
18	0	0	2	9	45	52	1.4520	0.8687	1.4443	0.8560	0.53	1.48
19	−2	0	0	5	45	40	0.2233	0.1724	0.2187	0.1579	2.10	9.21
20	0	−2	0	9	35	40	0.3029	0.1916	0.2998	0.1812	1.03	5.72

Note: *p* < 0.05 is significant, *p* < 0.01 is highly significant, and *p* > 0.05 is not significant.

**Table 3:** ANOVA results of the fitted quadratic regression models.

Source	Response A_1_				Response A_2_			
	Sum of Squares	Mean Square	F-Value	*p*-Value Probability > F	Sum of Squares	Mean Square	F-Value	*p*-Value Probability > F
Model	2.05	0.23	5498.52	<0.0001 significant	0.69	0.076	353.71	<0.0001 significant
*x*_1_-Cycle number	0.058	0.058	1409.22	<0.0001	0.015	0.015	69.31	<0.0001
*x*_2_-Output level	0.030	0.03	724.18	<0.0001	0.014	0.014	63.49	<0.0001
*x*_3_-Gain (dB)	1.61	1.61	38998	<0.0001	0.55	0.55	2538.12	<0.0001
*x*_1×2_	1.54 × 10^−4^	1.54 × 10^−4^	3.72	0.0827	2.03 × 10^−3^	2.03 × 10^−3^	9.38	0.0120
*x*_1×3_	0.019	0.019	469.29	<0.0001	2.45 × 10^−3^	2.45 × 10^−3^	11.32	0.0072
*x*_2×3_	7.29 × 10^−3^	7.29 × 10^−3^	176.04	<0.0001	2.90 × 10^−3^	2.90 × 10^−3^	13.42	0.0044
*x*_1_^2^	3.44 × 10^−3^	3.44 × 10^−3^	83.01	<0.0001	1.21 × 10^−3^	1.21 × 10^−3^	5.58	0.0397
*x*_2_^2^	2.54 × 10^−8^	2.54 × 10^−8^	6.15 × 10^−4^	0.9807	7.61 × 10^−5^	7.61 × 10^−5^	0.35	0.5660
*x*_3_^2^	0.28	0.28	6776.11	<0.0001	0.09	0.09	415.42	<0.0001
Residual	4.14 × 10^−4^	4.14 × 10^−5^			2.16 × 10^−3^	2.16 × 10^−4^		
Lack of Fit	2.76 × 10^−4^	5.53 × 10^−5^	2.01	0.2314; not significant	1.53 × 10^−3^	3.06 × 10^−3^	2.44	0.1752; not significant
Pure Error	1.38 × 10^−4^	2.75 × 10^−5^			6.28 × 10^−4^	1.26 × 10^−4^		
Cor Total	2.05				0.69			

**Table 4:** Error statistical analysis of the regression equations.

Response	Standard Deviation	Mean	C.V. (%)	PRESS	R-Squared	Adjusted R-Squared	Predicted R-Squared	Adeq. Precision
A_1_	6.435 × 10^−3^	0.46	1.39	2.51 × 10^−3^	0.9998	0.9996	0.9988	286.028
A_2_	0.015	0.29	5.11	0.014	0.9969	0.9941	0.9803	73.321

**Table 5:** Comparison of the predicted values with the RSM and the experimental values.

Cycle Number	Output Level	Gain	Actual Values		Predicted Values		Relative Errors		
			A_1_ (V)	A_2_ (V)	A_1_′ (V)	A_2_′ (V)	*δ*_A1_ (%)	*δ*_A2_ (%)	Δ*β*′ (%)
8	36	50	0.9396	0.5967	0.9706	0.5825	−3.20	1.39	8.20
8	36	44	0.4759	0.3207	0.4854	0.3059	−1.96	4.46	8.67
10	52	50	1.5298	0.9222	1.4118	0.8423	8.36	9.45	−6.78
10	48	44	0.7148	0.4355	0.7281	0.4453	−1.83	−2.24	1.44
10	46	42	0.5480	0.3394	0.5505	0.3408	−0.46	−0.25	0.68
10	38	42	0.4642	0.286	0.4617	0.2707	0.54	7.05	5.91
8	54	30	0.1463	0.075	0.1417	0.0672	3.25	−-3.05	−9.05
12	38	44	0.6108	0.3254	0.6607	0.3542	−7.55	−6.75	9.11
6	36	44	0.3759	0.3003	0.3767	0.2755	−0.20	7.43	7.86
10	36	42	0.4427	0.2500	0.4395	0.2520	0.72	1.01	−0.43

## Data Availability

No new data were created or analyzed in this study. Data sharing is not applicable to this article.
